# Integrated genomic analysis of antibiotic resistance and virulence determinants in invasive strains of *Streptococcus pneumoniae*

**DOI:** 10.3389/fcimb.2023.1238693

**Published:** 2023-10-19

**Authors:** Lin Liu, Yanfei Wang, Lihong Ge, Dongping Hu, Xi Xiang, Ying Fu, Jun Lu, Xi Li, Yunsong Yu, Yuexing Tu, Xueqing Wu

**Affiliations:** ^1^Laboratory Medicine Center, Department of Clinical Laboratory, Zhejiang Provincial People’s Hospital, Affiliated People’s Hospital, Hangzhou Medical College, Hangzhou, Zhejiang, China; ^2^Department of Infectious Diseases, Sir Run Run Shaw Hospital, Zhejiang University School of Medicine, Hangzhou, Zhejiang, China; ^3^Sir Run Run Shaw Hospital, Zhejiang University School of Medicine, Key Laboratory of Microbial Technology and Bioinformatics of Zhejiang Province, Hangzhou, Zhejiang, China; ^4^Regional Medical Center for National Institute of Respiratory Diseases, Sir Run Run Shaw Hospital, Zhejiang University School of Medicine, Hangzhou, Zhejiang, China; ^5^Department of Clinical Laboratory, The Children’s Hospital, Zhejiang University School of Medicine, National Clinical Research Center for Child Health, Hangzhou, Zhejiang, China; ^6^Department of Infectious Disease, Affiliated Dongyang Hospital of Wenzhou Medical University, Dongyang, Zhejiang, China; ^7^Department of Clinical Laboratory, Affiliated Jinhua Hospital, Zhejiang University School of Medicine, Jinhua, Zhejiang, China; ^8^Department of Clinical Laboratory, Sir Run Run Shaw Hospital, School of Medicine, Zhejiang University, Hangzhou, Zhejiang, China; ^9^Key Laboratory of Precision Medicine in Diagnosis and Monitoring Research of Zhejiang Province, Hangzhou, Zhejiang, China; ^10^The Quzhou Affiliated Hospital of Wenzhou Medical University, Quzhou People’s Hospital, Quzhou, Zhejiang, China; ^11^Department of Critical Care Medicine, Tongde Hospital of Zhejiang Province, Hangzhou, China

**Keywords:** invasive pneumococcal serotype, whole-genome sequencing, antibiotic resistance, rrgC, ZmpC

## Abstract

**Introduction:**

*Streptococcus pneumoniae* is an important human pathogen that may cause severe invasive pneumococcal diseases (IPDs) in young children and the elderly. A comprehensive comparative whole-genome analysis of invasive and non-invasive serotype strains offers great insights that are applicable to vaccine development and disease control.

**Methods:**

In this study, 58 invasive (strains isolated from sterile sites) and 71 non-invasive (serotypes that have not been identified as invasive in our study) pneumococcal isolates were identified among the 756 pneumococcal isolates obtained from seven hospitals in Zhejiang, China (2010–2022). Serotyping, antimicrobial resistance tests, and genomic analyses were conducted to characterize these strains.

**Results and discussion:**

The three most invasive serotypes were 23F, 14, and 6B. The invasive pneumococcal isolates' respective resistance rates against penicillin, ceftriaxone, tetracycline, and erythromycin were 34.5%, 15.5%, 98.3%, and 94.7%. Whole-genome sequencing indicated that the predominant invasive clonal complexes were CC271, CC876, and CC81. The high rate of penicillin non-susceptible *Streptococcus pneumoniae* (PNSP) is related to the clonal distribution of resistance-conferring penicillin-binding proteins (PBP). Interestingly, we found a negative correlation between invasiveness and resistance in the invasive pneumococcal serotype strains, which might be due to the proclivity of certain serotypes to retain their β-lactam resistance. Moreover, the mutually exclusive nature of *zmpC* and *rrgC+srtBCD *suggests their intricate and potentially redundant roles in promoting the development of IPD. These findings reveal significant implications for pneumococcal vaccine development in China, potentially informing treatment strategies and measures to mitigate disease transmission.

## Introduction

*Streptococcus pneumoniae* is an important pathogen that causes invasive pneumococcal diseases (IPDs) such as invasive pneumonia, sepsis, bacteremia, and meningitis (Weiser et al., 2018). The most infected population comprises young children, older adults, and immunocompromised patients (O’Brien et al., 2009). IPDs can be effectively controlled by introducing pneumococcal conjugate vaccines (PCVs) which are designed by target the most prevalent and virulent pneumococcal serotypes (Whitney et al., 2003; O’Brien et al., 2009; Pneumococcal Disease: Prevention | CDC, 2023). Epidemiological studies of IPD have consistently reported specific serotypes that cause invasive diseases, such as the recently reported serotype 4 and 24F IPD emergence in Israel and France, respectively (Kellner et al., 2021; Lo et al., 2022). To identify important virulent strains, a comparative investigation between isolates from different origins is widely accepted (Sharew et al., 2021; Yan et al., 2021). However, there is a potential risk of overlooking important differences between invasive and non-invasive serotype strains. Investigations of this type appear to be relatively scarce in the current literature, which are crucial for understanding the disease-causing potential of invasive pneumococcal serotypes and for contributing to vaccination strategies and vaccine development.

The selection of anti-infective treatment regimens should consider the drug resistance status of the strain. The increasing resistance of *S. pneumoniae* to β-lactams, macrolides, and tetracyclines has led to increasingly limited options for treating IPD (Thummeepak et al., 2015; Suaya et al., 2020). The high resistance of *S. pneumoniae* to macrolides and tetracyclines in China means that these drugs are seldom prescribed, and β-lactams are the first-line treatment for IPDs (Zhou et al., 2022b). However, macrolides are still recommended for regions with resistance rates below 25% (Gregory and Davis, 2020), and the global increase in the non-susceptibility *S. pneumoniae* to β-lactams highlights the critical need to monitor the antibiotic resistance status of invasive pneumococci.

The development of IPD usually starts with bacterial upper respiratory colonization, in which pneumococcus may asymptomatically colonize the host (Bogaert et al., 2004). If pneumococcal strains evade the host immune defense and reach the lower respiratory tract, they can cause inflammation and fluid accumulation in the lungs, which is normally confirmed as pneumonia (Coonrod, 1989; Bogaert et al., 2004). Once the pneumococcus spreads to the bloodstream or cerebrospinal fluid, it causes sepsis or meningitis, which can lead to organ failure and death (Mook-Kanamori et al., 2011; Weiser et al., 2018). During this process, various pneumococcal virulence factors contribute to immune evasion (capsule polysaccharides, Cps), epithelial cell adhesion (pneumococcal *rrg* pathogenic island), and host tissue invasion (zinc metalloproteinase, ZmpC) (Jonsson et al., 1985; Camilli et al., 2006; El Mortaji et al., 2012; Cremers et al., 2014). Comparative whole-genome sequencing (WGS) analysis of virulence factors among invasive and non-invasive serotype strains, for example the presence of critical virulence genes in invasive isolates rather than non-invasive serotype strains, would be a valuable contribution to our understanding of IPD development.

In this study, we identified 58 invasive pneumococcal strains which were isolated form sterile site from a pool of 756 isolates collected from seven hospitals in Zhejiang, China, during the period 2010–2022. Those 58 invasive pneumococcal cover 16 serotypes. A genomic comparison analysis was conducted between these strains and a set of 71 strains from serotypes that had not been previously identified as invasive in our collected cases. This study aimed to investigate the serotype distribution, antimicrobial susceptibility, molecular epidemiology, and virulence factors of invasive pneumococcal serotype strains in Zhejiang Province, China.

## Materials and methods

### *Streptococcus pneumoniae* isolation and serotyping

*S. pneumoniae* isolates (n=129) were collected from seven tertiary hospitals in Zhejiang, China, from July 2010 to January 2022, and included 58 invasive and 71 non-invasive serotype strains. Invasive isolates were collected from patients’ sterile site, for instance, blood, bronchoalveolar lavage fluid (BALF), and cerebrospinal fluid (CSF) specimens. Non-invasive serotype isolates were those strains obtained from sputum, nasopharynx, and oropharynx specimens and belong to serotypes that have never been identified in IPDs in all of our pneumococcal-positive cases. All isolates were obtained by culturing the clinical samples on blood agar plates at 37°C with 5% CO_2_ and were identified by optochin, bile solubility, and *lytA* PCR tests. Thereafter, all isolates were subjected to serotyping by the latex agglutination test and Quellung reaction (SSI Diagnostica, Denmark). We also conducted *in silico* serotyping after WGS (detailed below) using SeroBA software (https://github.com/sanger-pathogens/seroba) (Epping et al., 2018).

### Clinical information collection

The clinical information of all patients in each IPD case was retrospectively extracted from their medical records with the approval of the Sir Run Shaw Hospital Ethics Review Committee (Zhejiang University School of Medicine, 20201112-32), which included data on sex, age, and primary diagnosis (**Table 1**). The specimen types of all cases are summarized in **Table 2** (IPD cases) and **Supplementary Table 1** (non-invasive pneumococcal infection cases).

**Table 1:** Demographic and clinical characteristics of IPD patients.

Characteristics	Prospective	
	No. of patients	%
**Total**	58	100.0
**Gender**		
Male	40	69.0
Female	18	31.0
**Age(years)**		
0-5	26	44.8
6-64	21	36.2
≥65	11	19.0
**Primary diagnosis**		
Meningitis	6^*^	10.3
Sepsis	7	12.1
Bacteremia	1	1.7
Pneumonia	16^*^	27.6
Bronchitis	3	5.2
URTI	4	6.9
Other respiratory disease^a^	4	6.9
Unexplained fever	7	12.1
Trauma	2	3.4
Others^b^	9	15.5

URTI, upper respiratory tract infection.

‘a’, Asthma, COPD, Tonsillitis; ‘b’, systemic lupus erythematosus, lung cancer, cerebrospinal fluid otorrhea, nephrotic syndrome, acute enteritis, unexplained headache; ‘*’, a patient diagnosed with both meningitis and pneumonia.

**Table 2:** Invasive specimen type and related pneumococcal diseases.

Specimen type	Prospective		
	No. of specimen		%
**All**	58		100.0
**Blood**			
Meningitis	2		3.4
Sepsis	7		12.1
Bacteremia	1		1.7
Pneumonia	10		17.2
Bronchitis	3		5.2
URTI	4		6.9
Other respiratory disease^a^	4		6.9
Unexplained fever	7		12.1
Trauma	2		3.4
Others^b^	9		15.5
Total	49		84.5
**CSF**			
Meningitis	4		6.9
Pneumonia	1		1.7
Total	4*		6.9
**BLAF**			
Pneumonia	5		8.6
Total	5		8.6

CSF, cerebrospinal fluid; BLAF, bronchoalveolar lavage fluid.

URTI, upper respiratory tract infection.

‘a’, Asthma, COPD, Tonsillitis; ‘b’, systemic lupus erythematosus, lung cancer, cerebrospinal fluid otorrhea, nephrotic syndrome, acute enteritis, unexplained headache; ‘*’, a patient diagnosed with both meningitis and pneumonia.

### Antimicrobial susceptibility test

Broth microdilution assays were used to determine the minimal inhibitory concentrations (MICs) of the tested antimicrobial agents according to the Clinical and Laboratory Standard Institute (CLSI) protocols, as described previously (Lewis, 2023). The antimicrobial agents tested were penicillin (PEN), ceftriaxone (CRO), erythromycin (ERY), and tetracycline (TET). *S. pneumoniae* strain ATCC 49619 was used as the quality control strain. The results were defined according to the 2023 Clinical and CLSI Guidelines M100-Ed33 (Lewis, 2023).

### WGS and analysis

Genomic DNA was extracted from all isolates using a QIAamp® DNA Mini Kit (Qiagen, Valencia, CA, USA) and sequenced using an Illumina HiSeq X 10 platform (Illumina, San Diego, CA, USA). The Illumina reads were assembled by end pairing using Shovill (Seemann T, https://github.com/tseemann/shovill), with a minimum splicing length of 200 bp and a minimum coverage of 10-fold. The final assemblies have the N50 not less than 60K and the minimum sequencing depth is 300X. *In silico* sequence type (ST) of each strain was then obtained by blasting our genome against the pubMLST database (https://pubmlst.org) via mlst (Seemann T, https://github.com/tseemann/mlst) (Jolley and Maiden, 2010). To confirm the serotyping results of our Quellung reactions, we also conducted *in silico* serotyping by comparing sequenced reads to a database containing key genes that determine serotypes in *cps* gene clusters using SeroBA. Virulence genes and antimicrobial resistance genes were screened using ABRicate software (Seemann T, Abricate, GitHub https://github.com/tseemann/abricate). Prominent amino acid substitutions in penicillin (PEN)-binding proteins (PBP1a, PBP2b, and PBP2x) were analyzed by BLST+ (2.13.0) (https://github.com/ncbi/blast_plus_docs) against the database of CDC, USA (https://www.cdc.gov/streplab/pneumococcus/mic.html). Phylogenetic trees were constructed using popPUNK (Lees et al., 2019) (https://github.com/bacpop/PopPUNK) and visualized using iTOL (v6, https://itol.embl.de).

### Statistical analysis

To assess the difference in antibiotic resistance between invasive and non-invasive isolates, we conducted a Mann–Whitney U test on each column, where a *p<*0.0001 was considered statistically significant. The correlation between invasive and PEN insensitivity ratios for each serotype was calculated using a two-tailed method. A Pearson correlation coefficient (r)<-0.7 was considered a strong negative correlation, r<-0.5 was considered a moderate correlation, and r<-0.3 was considered a weak correlation. A two-tailed *p*<0.05 was considered statistically significant. All analyses were performed using GraphPad Prism v9.5.0.

## Results

### Clinical characteristics of patients with IPD

The demographic and clinical characteristics of patients with IPD are summarized in **Tables 1**, **2**. Among 58 patients with IPD, males accounted for the majority (69.0%). In the different age groups, 44.8% (26/58) of IPD cases were identified in young children (0–5 years), and 19.0% (11/58) occurred in patients ≥65 years old. The three most common primary diagnoses were bronchitis, pneumonia, and sepsis. The specimen types used were blood (49/58, 84.5%), BALF (5/58, 8.6%), and cerebrospinal fluid (CSF, 4/58, 6.9%). Bronchitis, fever, and sepsis were the primary diagnoses of cases that later been confirmed as IPD due to pneumococcal blood culture positive. In cases of non-invasive pneumococcal infection, the most commonly primary diagnosed disease was pneumonia (**Supplementary Table 1**).

### Serotype distribution

**Figure 1:**
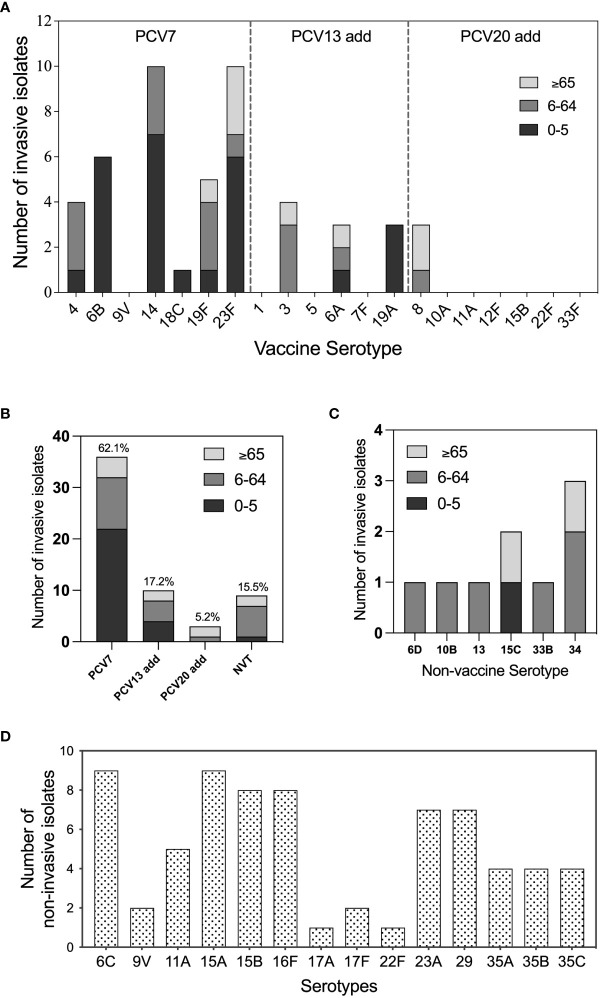
Serotype distribution and vaccine serotype coverage of pneumococcal isolates All tested *S. pneumoniae* isolates were serotyped by quellung reactions and *in silico* by whole genome sequencing via SeroAB. **(A)** The distribution of invasive pneumococcal vaccine serotypes in different age groups: 0-5 (blank), 6-65 (grey), and, >65 (light grey); **(B)** The proportion of pneumococcal conjugate vaccine (PCV) 7, PCV13-add, PCV20-add, and non-vaccine serotype (NVT) invasive pneumococcal isolates; **(C)** Serotype distribution of NVT invasive pneumococcal isolates; **(D)** Serotype distribution of pneumococcal isolates belong to the serotypes that are never been detected for invasive cases (non-invasive serotypes).

Among all invasive strains, 16 serotypes were identified, including 10 vaccine serotypes and six non-vaccine serotypes, which accounted for 84.5% (49/58) and 15.5% (9/58) of all invasive isolates, respectively (**Figures 1A, B**). The top three invasive serotypes were 23F (17.2%, 10/58), 14 (17.2%, 10/58), and 6B (10.3%, 6/58), all of which were covered by 7-valent pneumococcal polysaccharide conjugate vaccine (PCV7) (**Figure 1A**). The most frequently isolated non-vaccine-invasive serotypes were 34 and 15C (**Figure 1C**). Young children were the most frequently infected population with IPD, wherein especially the serotypes 6B, 14, and 23F caused IPD cases. We also included 71 non-invasive pneumococcal serotype strains in our study. The serotype distribution data showed that 6C, 15A, 15B, and 16F were the most frequently identified serotypes in cases of non-invasive pneumococcal infection (**Figure 1D**).

### Antimicrobial susceptibility

Antimicrobial susceptibility results for the 58 invasive *S. pneumoniae* isolates are presented in **Table 3**. According to the non-meningitis breakpoint, the non-susceptibility rates of the isolates to PEN, CRO, TET, and ERY were 34.5%, 15.5%, 98.3%, and 94.7%, respectively. Regarding the meningitis breakpoint, the insensitivity rates of the isolates against PEN and CRO were 82.8% and 43.1%, respectively. Most PEN- and CRO-non-susceptible strains were PCV-covered serotypes, whereas serotypes 19F and 14 accounted for the majority. The MIC values of each isolate are presented in **Supplementary Tables 3** and **4**, showing that the MIC90 values of invasive and non-invasive pneumococcal isolates against PEN were 8 and 4 μg/mL, respectively.

**Table 3:** Antibiotic susceptibility of invasive *S. pneumoniae*.

Serotype	Total (%)	Proportion (%) of non-susceptible isolates against:					
		PEN^a^	PEN^b^	CRO^a^	CRO^b^	TET	ERY
**PCV7**							
4	4(7.0)	0	0	0	0	100	50
6B	6(10.5)	50	100	0	66.7	83.3	100
14	10(15.8)	66.7	100	10	55.6	100	100
18C	1(1.8)	100	100	100	100	100	100
19F	5(8.8)	80	100	100	100	100	100
23F	10(17.5)	40	100	10	50	100	100
**PCV13 add**							
3	4(7.0)	0	50	0	0	100	100
6A	3(5.3)	33.3	100	0	66.7	100	100
19A	3(5.3)	33.3	100	33.3	66.7	100	100
**PCV20 add**							
8*	2(3.5)	0	50	0	0	100	100
**NVT**							
6D	1(1.8)	0	100	0	0	100	100
10B	1(1.8)	0	0	0	0	100	100
13	1(1.8)	0	100	0	0	100	100
15C	2(3.5)	0	100	0	50	100	100
33B	1(1.8)	0	100	0	0	100	100
34	3(5.3)	0	66.7	0	0	100	66.7
**Total (%)**	57*	34.5	82.8	15.5	43.1	98.3	94.7

^a^Non-meningitis breakpoint: PEN resistant breakpoint, S ≤ 2 ug/ml, I = 4 ug/ml, R ≥ 8 ug/ml; CRO resistant breakpoint, S ≤ 1 ug/ml, I = 2 ug/ml, R ≥ 4 ug/ml.

^b^Meningitis breakpoint: PEN resistant breakpoint, S ≤ 0.06 ug/ml, R ≥ 0.12 ug/ml; CRO resistant breakpoint, S ≤ 0.5 ug/ml, I = 1 ug/ml, R ≥ 2 ug/ml.

*, one serotype 8 strain had only a blood sample and could not be tested for antimicrobial susceptibility

PEN, Penicillin; CRO, Ceftriaxone; TET, Tetracycline; ERY, Erythromycin.

### Phylogenetic analysis and antimicrobial resistance gene determination

**Figure 2:**
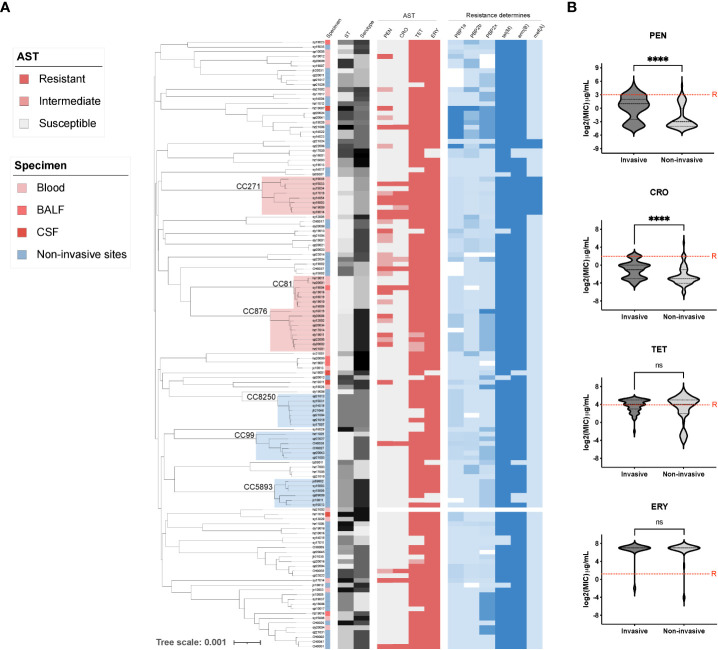
The phylogenetic tree and antibiotic resistance determinants of invasive and non-invasive serotype pneumococcal strains. **(A)** The phylogenetic tree of all tested pneumococcal isolates (n=129) was constructed in PopPUNK, where three major clone complexes (CC) for invasive and non-invasive serotype isolates were shaded in light red and blue, respectively. The metadata including specimen types, sequence type (ST), serotype, and antibiotic susceptibility test results (AST) was aligned for all sequenced pneumococcal isolates, which was followed by the detection of antibiotic resistance determinants of PBPs, *tetM, ermB,* and *mefA*; **(B)** Minimal inhibition concentration (MIC) comparison between invasive and non-invasive serotype strains. “****” indicate a significant difference with a p-value less than 0.0001, “ns” indicate no significance was detected, red dash line is the cut-off of resistant MIC.

To further understand the genetic characteristics of the invasive *S. pneumoniae* strains, we conducted WGS analyses of all invasive pneumococcal isolates (n=58) and compared them with the non-invasive isolates (n=71). According to our phylogenetic analysis (**Figure 2A**), the most prevalent invasive clone clusters (CCs) were CC876 (15.5%, 9/58, serotype 14), CC271 (13.8%,8/58, serotype 19F/19A), and CC81 (12.1%, 7/58, serotype 23F). The major non-invasive *S. pneumoniae* clones were CC8250 (9.9%, 7/71, serotype 16F), CC99 (8.5%, 6/71, serotype 11A/35A), and CC5893 (8.5%, 6/71, serotype 29). The beta-lactam non-sensitive strains were mainly distributed in the invasive branches and were concentrated in the prevalent clones CC271, CC876, and CC81, which carry PBP1a-2b-2x types of 13-11-33, 60-16-312, and 15-12-18, respectively. Correspondingly, a comparison of MIC values between invasive and non-invasive strains showed significantly higher MICs in invasive pneumococcus against PEN and CRO (**Figure 2B**). We also identified a novel PBP2x type in strain dy19012 (ST342, serotype 23F) and a novel PBP2b type in strain sy19004 (ST81, serotype 23F) that may mediate PEN resistance (MIC=8 μg/mL). Regardless of their invasiveness, all strains presented high-level resistance against ERY and TET; most carried genes of *ermB* and *tetM*, and the carry rate for having both *ermB* and *tetM* is 90.6% (116/128). Another macrolide resistance-encoding gene, *mef(A)* was prevalent mainly in CC27, and the carry rate for having both mefA and tetM is 7.8% (10/128). The genome of hz21002 was obtained directly by sequencing the blood sample and did not pass quality control, resulting in unavailability of data for this isolate.

### Correlation of beta-lactam resistance and invasion

**Figure 3:**
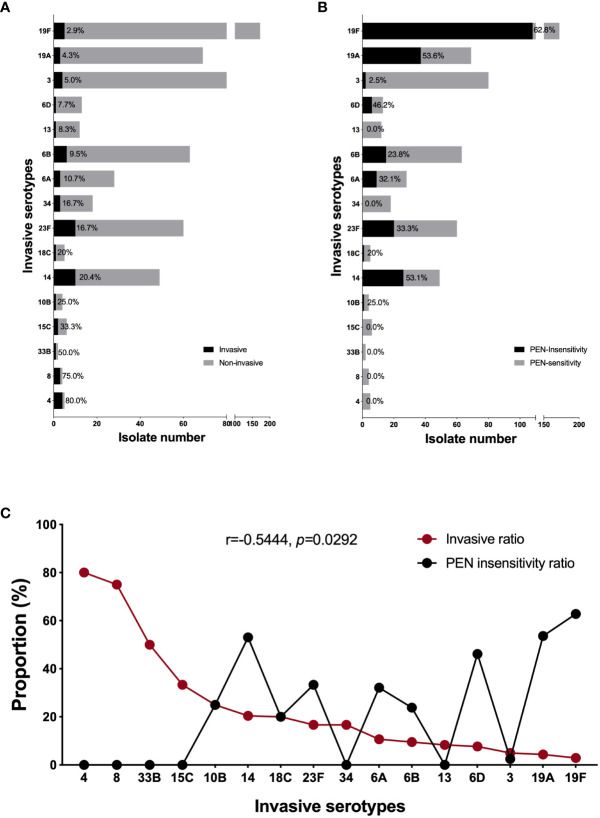
The correlation between invasiveness and penicillin non-susceptibility of invasive serotypes. The proportion of invasive **(A)** and PEN non-susceptible **(B)** isolate in each invasive serotype (included all our strain bank isolates, n=756); **(C)** The correlation analysis between the invasiveness and PEN non-susceptibility in all invasive serotypes. A Pearson correlation coefficient (r) <-0.7 was considered a strong negative correlation, r<-0.5 was considered a moderate correlation, and r<-0.3 was considered a weak correlation. A two-tailed p<0.05 was considered statistically significant.

Careful analysis of the above datasets and taking into account our total pneumococcal strain storage (n=756) revealed that the serotype 19F strains presented the highest PEN insensitivity rate of 62.8% (108/172) and the lowest invasive ratio of 2.9% (5/172). In contrast, 80% (4/5) of the serotype 4 strains in our study were invasive isolates and none were PEN-insensitive (**Figures 3A, B**). This finding prompted us to perform correlation analysis for all invasive serotypes. As shown in **Figure 3C**, the invasive and PEN insensitivity ratios of the invasive serotypes were negatively and moderately correlated, respectively (r=-0.5444, *p*=0.292). Among all invasive serotypes, 19F and 19A were the most resistant and least invasive, while serotypes 4 and 8 were the most invasive and least resistant, respectively. Because 19F is the most prevalent strain in our study resulting in its high isolation rate in the invasive strains, we conducted a separate analysis that included all available genome data of serotype 19F strains (n=124) in our laboratory. As shown in **Supplementary Figure 1**, most serotype 19F isolates belonged to serotype CC271. The PEN insensitivity of this serotype is very high (62.8%), and is mediated by the same PBP1a-2b-2x type (13-11-33). Among more than hundred 19F strains, only five were invasive.

### Virulence factors analysis

**Figure 4:**
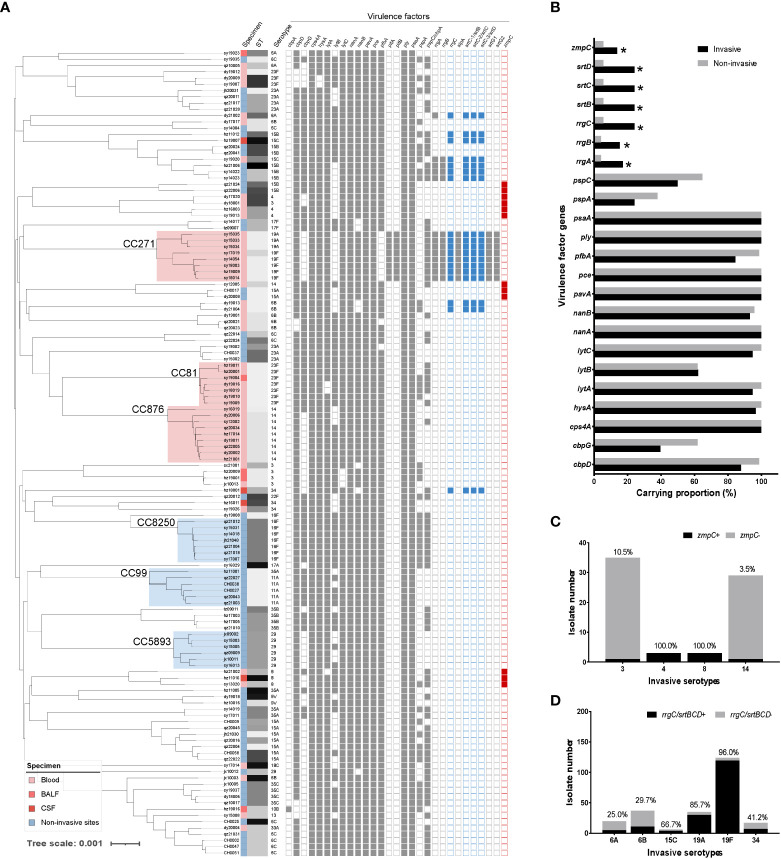
The virulence factor analysis of invasive and non-invasive serotype pneumococcal strains. **(A)** The detection of virulence factors was attached for each strain in the phylogenetic tree of invasive and non-invasive serotype strains. **(B)** Carrying proportion of virulence factors for invasive (black) and non-invasive (grey) serotype strains. Several virulence factors are clonally distributed (*cbpA* and *pitAB*.) or carried by all strains (*ply* and *psaA*), which were excluded from the analysis. **(C)** Distribution and proportion of zmpC in the carried serotypes of 3, 4, 8, and 14; D. Distribution and proportion of rrgC+srtBCD in the carried serotypes of 6A, 6B, 15C, 19A, 19F, and 34.

To determine the virulence of invasive pneumococci, we conducted virulence gene screening for both invasive and non-invasive strains. As shown in **Figure 4**, the choline-binding protein gene *cbpA* was present in only one strain. The virulence factor-encoding genes *pitAB* (iron uptake) and *srtG1,2* (cognate sortase) were only present in the CC271 strains. The genes *cpsA* (capsule synthesis), *hysA* (hyaluronidase), *lytABC* (autolysin), *nanAB* (neuraminidase), *pavA* (fibronectin-binding protein), *pce* (phosphorylcholine esterase), *ply* (pneumolysin), and *psaA* (pneumococcal surface adhesin A) were present in both invasive and non-invasive strains with no clone specificity. The non-invasive strains carried slightly more *cbpGD*, *pfbA* (plasmin- and fibronectin-binding protein A), and *pspAC* (pneumococcal surface protein A) genes. Moreover, the genes *rrgABC* (pilus adhesin), *srtBCD* (sortase), and *zmpC* (zinc metalloproteinase) tended to be carried by invasive strains. Interestingly, the co-occurrence of *zmpC* and *rrgC+srtBCD* was mutually exclusive, and *zmpC* was mainly carried by serotypes 4 and 8, whereas *rrgC+srtBCD* appeared more frequently in the 19F and 19A strains. Again, the analysis targeting only serotype 19F showed *rrgABC+srtBCD* was clonally distributed in these isolates (**Supplementary Figure 2**).

## Discussion

The identification of prevalent invasive serotypes is crucial for effective prevention and management of patients with IPD. The data presented in this study indicate that the most prevalent IPD serotypes in Zhejiang, China, are 23F, 14, and 6B. However, the CSF-isolated pneumococcal serotypes were 34, 8, and 15C, of which 34 and 15C were the most commonly identified non-vaccine serotypes. Owing to the low PCV coverage in China, the prevalence of IPD serotypes is different from that in developed countries (Pick et al., 2020; Suaya et al., 2020; Yanagihara et al., 2021; Wu et al., 2022). A recent multicenter study of 300 invasive *S. pneumoniae* isolates predominantly from northern China indicated that the most prevalent invasive serotypes are 23F, 19F, and 19A, and the top CSF-isolated serotypes are 23F, 19F, and 14 (Zhou et al., 2022a). A similar study from western China showed that the most prevalent pneumococcal serotypes are 19F, 19A, and 6B (Yan et al., 2021). The inconsistent results found in the literature as well as in our study highlight the variation in serotype distribution across different regions of China. Therefore, it is crucial to conduct a national surveillance study on invasive *S. pneumoniae* to accurately assess this situation. The region-specific dominance of certain serotypes may direct different vaccine strategies. While PCV13 and PCV20 cover the majority of IPD serotypes in Zhejiang Province, non-vaccine serotypes 34 and 15C have caused highly virulent cases of meningitis that require continued vigilance.

Our AST results showed that the nonsusceptibility to penicillin and ceftriaxone in IPD isolates was significantly higher than that in non-invasive serotype strains. But further analysis indicates that β-lactams resistance was mainly mediated by the clonal distribution of CC271, CC876, and CC81 strains carrying certain PBP1a-2b-2x combinations which were confirmed as PEN-non-susceptible PBP types from a global pneumococcal database (Li et al., 2016). For this reason, according to the non-meningitis breakpoint, we reported a very high PNSP rate of over 30% for invasive *S. pneumoniae* tested in the current study. A study conducted in China that collected 993 strains of *S. pneumoniae* up to 2017 showed that the non-susceptibility rate of IPD strains to penicillin reached 22.4%, which was significantly higher than that of the non-invasive strains (Yan et al., 2021). However, another pneumococcal epidemiological study collected 300 invasive pneumococci from 2010 to 2015 and reported a very low PNSP rate (4.3%) (Zhou et al., 2022b). Later, the same group performed a deeper analysis of PBPs using conventional PCR and showed that the PBP site substitutions in PNSP strains matched our findings of non-susceptible PBP, which were distributed mostly in prevalent clones. Although *in silico* predicted MIC of strains carrying non-susceptible PBPs against penicillin was 4 μg/mL (Li et al., 2016), most of the isolates in their study had an MIC of 1–2 μg/mL. The reason for these inconsistent results might be due to MIC value interpretation, sample collection time periods, and regional differences. Macrolide and tetracycline resistance was maintained at high levels in both invasive and non-invasive pneumococci, which is mediated by the national distribution of *ermB* and *tetM* in China (Zhou et al., 2022b), indicating the limited clinical value of such drugs in China.

Notably, some serotype strains had very high invasive rates, but none of them presented a penicillin-resistant phenotype; therefore, we performed further analysis to examine the relationship between invasiveness and penicillin resistance. Among all the collected strains of invasive serotypes, we found a negative correlation between invasive and penicillin non-susceptibility rates. To date, no relevant reports have been published regarding *S. pneumoniae*. However, in the gram-negative bacterium *Klebsiella pneumoniae*, hypervirulent strains have been reported to exhibit a relatively low ability to acquire antibiotic-resistant plasmids and hardly simultaneously exhibit virulence and resistance (Tian et al., 2022). An ongoing study in our laboratory has shown that serotype 19F strains display greater proclivity to maintain their β-lactam resistance phenotype than that of other strains. In the top three invasive serotypes (4, 8, 33B), none of the strains to been found resistant to PEN in our study. Recent reports demonstrated the same PSSP for 190 invasive serotype 4 strains and 90 invasive serotype 8 strains (Hansen et al., 2021; Kellner et al., 2021). No report of PEN resistance was found in pneumococcal serotype 33B strains. The mechanism behind such phenotype need further identification. The negative correlation observed between the invasiveness and non-susceptibility of *S. pneumoniae* may be attributed to the presence and retention of specific virulence factors and resistance determinants in the highly invasive and resistant serotype strains, respectively. A more comprehensive understanding of this newly discovered epidemiological phenomenon requires further investigation into its underlying mechanisms.

During the invasive infection process, various virulence factors participate at different stages. We did not find a carrying difference between invasive and non-invasive pneumococcal serotype strains in several classical virulence factor-encoding genes, such as capsule synthesis, pneumococcal surface adhesin, autolysin, fibronectin-binding protein, and pneumolysin, which are similar to those reported previously (Yan et al., 2021). However, *rrgABC* (pilus adhesin), *srtBCD* (sortase), and *zmpC* (zinc metalloproteinase) are mostly carried by invasive pneumococci, and the *rrgABC+srtBCD* locus is clonally distributed in CC271 strains. There are two intriguing points in this section regarding these results. First, except for CC271, one of the pilus type 1 (P1) encoding genes, *rrgC*, would be independently expressed in the invasive strains together with the three P1 specific sortase encoding genes, *srtBCD*. RrgC has been reported as a lectin targeting different host glycosylations; however, it is the least understood pilus protein in *S. pneumoniae* (Day et al., 2017). Our findings demonstrate the importance of sortase for pilus expression and the small pili RrgC for *S. pneumoniae* causing invasive disease. Future pneumococci-host interaction studies would be valuable by focusing on these virulence factors. Furthermore, *rrgABC+srtBCD* was initially identified in the highly invasive serotype 4 strain TIGR4 (Barocchi et al., 2006). However, none of our serotype 4 invasive isolates was found to carry P1-related genes; instead, they encoded a zinc metalloproteinase. The co-occurrence of *zmpC* and *rrgC+srtBCD* was mutually exclusive, and *zmpC* was mainly carried by serotypes 4 and 8, which are highly correlated with IPDs. A study conducted in Italy (Camilli et al., 2006) demonstrated that serotypes 8 and 11A carried *zmpC*, and another study from the Netherlands (Cremers et al., 2014) revealed that *zmpC* was predominantly associated with serotypes 8, 4, 33A/F, and 11A/D. In contrast, our laboratory isolates of serotype 11A were collected from patients without IPD and no *zmpC* was detected in these strains. Pneumococcal ZmpC is involved in the breakdown of host tissues, whereas the pilus biogenesis proteins RrgC and SrtBCD play a role in the adhesion and colonization of the bacterium. There is no clear explanation for the mutual exclusion of these two different virulence factors; they may interfere with the expression of other functions or they are carried on different mobile genetic elements that are not transferable between different clones. Nevertheless, our findings demonstrate that both are important for the invasiveness of different invasive pneumococcal serotypes. Research on the serotype-specific virulence mechanism of *S. pneumoniae* would be significantly more meaningful than studying its overall pneumococcal virulence.

In conclusion, this study provides valuable insights into invasive *S. pneumoniae* serotypes and their management. A national surveillance study is necessary to understand the variation in invasive serotype distribution across different regions of China, which is crucial for PCV vaccination strategies. A high PNSP rate was related to the clonal distribution of non-susceptible PBP types, and we found a negative correlation between invasiveness and resistance in invasive pneumococcal strains. The mutually exclusive nature of *zmpC* and *rrgC+srtBCD* suggests their intricate and potentially redundant roles in promoting the development of IPD. Further mechanistic studies will contribute to the development of pneumococcal vaccines.

## Data availability statement

The datasets presented in this study can be found in online repositories. The names of the repository/repositories and accession number(s) can be found below: https://www.ncbi.nlm.nih.gov/genbank/, PRJNA977823 https://www.ncbi.nlm.nih.gov/genbank/, PRJNA795524 https://www.ncbi.nlm.nih.gov/genbank/,PRJNA924107.

## Author contributions

XW, YT and YY conceptualized and designed the study, drafted the initial manuscript, and reviewed and revised the manuscript. LL and YW contributed equally to the pneumococcal isolates collection, carried out the initial analyses, and reviewed and revised the manuscript. LG, DH, XX, JL, YF, and XL, contributed to clinical data collection, pneumococcal serotyping, and genomic sequencing, and reviewed and revised the manuscript. All authors contributed to the article and approved the submitted version.

## Supplementary material

Supplementary Figure 1 The phylogenetic tree and antibiotic resistance determinants of pneumococcal serotype 19F strains. The phylogenetic tree of all sequenced pneumococcal serotype 19F isolates (n=124) was constructed in PopPUNK, where the major clone complex (CC) 271 was shaded in light red. The metadata including specimen types, sequence type (ST), antibiotic susceptibility test results (AST), and PBPs types were aligned for all isolates.

Supplementary Figure 2 Virulence factor detection in serotype 19F strains. The detection of virulence factors was attached for all isolates in the phylogenetic tree along with specimen data and sequence type of pneumococcal serotype 19F strains (n=124). A similar virulence factor carrying pattern was noticed in this clone.
