# Supplementary material for: Integrated genomic analysis of antibiotic resistance and virulence determinants in invasive strains of Streptococcus pneumoniae
Source: Front Cell Infect Microbiol. 2023 Oct 19;13:1238693. doi: 10.3389/fcimb.2023.1238693 (PMC10620807; doi:10.3389/fcimb.2023.1238693)
Supplement: Supplementary file 1 [file Table_1.docx]

**Supplementary Table 1. Demographic and clinical characteristics of non-invasive pneumococcal infection patients**

| **Characteristics** | **Prospective** | |
| --- | --- | --- |
|  | **No. of patients** | **%** |
| **Total** | 70* | 100.0 |
| **Gender** |  |  |
| male | 42 | 60.0 |
| female | 28 | 40.0 |
| **Age(years)** |  |  |
| 0-5 | 45 | 64.3 |
| 6-64 | 10 | 14.3 |
| ≥65 | 15 | 21.4 |
| **Primary diagnosis** |  |  |
| Pneumonia | 35 | 50.0 |
| Bronchitis | 6 | 8.6 |
| URTI | 7 | 10.0 |
| Other respiratory disease^a^ | 3 | 4.3 |
| Fever | 1 | 1.4 |
| Trauma | 4 | 5.7 |
| Cancer | 5 | 7.1 |
| Others^b^ | 9 | 12.9 |

URTI, upper respiratory tract infection.

‘a’, chronic obstructive pulmonary disease, tonsillitis; ‘b’, chest tightness, nephritis, cerebral infarction, corneal ulcer, cardiac disease, uremia; ‘*’, one of 71 non-IPD patients with missing clinical data.

**Supplementary Table 2. The specimen type of 71 non-invasive strains.**

| **Specimen type** | **Prospective** | |
| --- | --- | --- |
|  | **No. of patients** | **%** |
| **Total** | 70* | 100.0 |
| **Sputum** |  |  |
| Pneumonia | 30 | 42.9% |
| Bronchitis | 5 | 7.1% |
| cancer | 5 | 7.1% |
| trauma | 3 | 4.3% |
| Other respiratory diseasea^a^ | 3 | 4.3% |
| Others^b^ | 7 | 10.0% |
| **Nasopharynx** |  |  |
| URTI | 6 | 8.6% |
| Pneumonia | 3 | 4.3% |
| Bronchitis | 1 | 1.4% |
| **Throat swabs** |  |  |
| Pneumonia | 2 | 2.9% |
| Fever | 1 | 1.4% |
| **Fester** |  |  |
| URTI | 1 | 1.4% |
| **Respiratory catheter** |  |  |
| Trauma | 1 | 1.4% |
| **Pus** |  |  |
| Corneal ulcer | 1 | 1.4% |
| **urine** |  |  |
| Nephritis | 1 | 1.4% |

URTI, upper respiratory tract infection.

‘a’, chronic obstructive pulmonary disease, tonsillitis; ‘b’, chest tightness, cerebral infarction, cerebral infarction, uremia, cardiac disease；‘*’, one of 71 non-IPD patients with missing clinical data.

**Supplementary Table 3.** The MIC value (mg/L) of invasive *S. pneumoniae* isolates against tested antibiotics.

| **Isolates ID** | **PEN** | **CRO** | **TET** | **ERY** |
| --- | --- | --- | --- | --- |
| jx10003 | 0.125 | 0.125 | 32 | >128 |
| jx10013 | 0.125 | 0.125 | 16 | >128 |
| qz10005 | 2 | 1 | 16 | >128 |
| hz11016 | <0.06 | 0.125 | 4 | >128 |
| sy12002 | 4 | 0.5 | 4 | >128 |
| sy12005 | 16 | 4 | 32 | >128 |
| sy13020 | 0.125 | 0.125 | 16 | >128 |
| sy14054 | 16 | 4 | 16 | >128 |
| sy15008 | 0.125 | 0.125 | 32 | >128 |
| sy15033 | 16 | 4 | 16 | >128 |
| sy15034 | 2 | 1 | 16 | >128 |
| sy15035 | 2 | 0.5 | 16 | >128 |
| sy16019 | 2 | 0.25 | 8 | >128 |
| hz16003 | <0.06 | 0.125 | 32 | >128 |
| hz16011 | 1 | 0.5 | 32 | >128 |
| sy17014 | 8 | 4 | 32 | >128 |
| sy17019 | 4 | 4 | 16 | >128 |
| hz17014 | 1 | 0.5 | 4 | >128 |
| sy18003 | 8 | 4 | 16 | >128 |
| sy18014 | 32 | 4 | 4 | >128 |
| sy18019 | 1 | 0.5 | 8 | >128 |
| hz18001 | <0.06 | 0.125 | 32 | 0.25 |
| sy19004 | 8 | 4 | 8 | >128 |
| sy19007 | 0.5 | 0.25 | 32 | >128 |
| sy19009 | 2 | 0.5 | 16 | >128 |
| sy19013 | <0.06 | 0.125 | 16 | >128 |
| sy19020 | 1 | 1 | 32 | >128 |
| sy19023 | 1 | 0.25 | 8 | >128 |
| sy19026 | 1 | 0.125 | 32 | 128 |
| hz19001 | 2 | 0.125 | 8 | >128 |
| hz19007 | 2 | 0.5 | 32 | >128 |
| hz19009 | 1 | 2 | 16 | >128 |
| hz19011 | 1 | 1 | 16 | >128 |
| hz19016 | <0.06 | 0.125 | 16 | >128 |
| hz20001 | 1 | 0.5 | 8 | >128 |
| hz20009 | <0.06 | 0.125 | 32 | >128 |
| hz21001 | 0.5 | 0.25 | 2 | 128 |
| hz21002* |  |  |  |  |
| sx21001 | <0.06 | 0.125 | 4 | 128 |
| dy17017 | 0.25 | <0.06 | 32 | >128 |
| dy17020 | <0.06 | <0.06 | 32 | 0.25 |
| dy18001 | <0.06 | <0.06 | 32 | 0.25 |
| dy19001 | 4 | 1 | 32 | 128 |
| dy19010 | 4 | 1 | 8 | >128 |
| dy19011 | 8 | 1 | 2 | >128 |
| dy19012 | 8 | 1 | 32 | >128 |
| dy19013 | 8 | 1 | 0.25 | >128 |
| dy19016 | 8 | 1 | 8 | >128 |
| dy20002 | 8 | 1 | 2 | >128 |
| dy20004 | 0.125 | <0.06 | 32 | 128 |
| dy20006 | 8 | 1 | 4 | >128 |
| dy20009 | 0.5 | 0.125 | 32 | >128 |
| dy21002 | 4 | 1 | 16 | >128 |
| dy21004 | 4 | 0.5 | 32 | >128 |
| qz20021 | 2 | 1 | 32 | >128 |
| qz20023 | 2 | 1 | 32 | >128 |
| qz20034 | 2 | 1 | 4 | >128 |
| qz22005 | 4 | 1 | 8 | >128 |
| **MIC50** | 2 | 0.5 | 16 | >128 |
| **MIC90** | 8 | 4 | 32 | >128 |
| **MIC range** | 0.06-32 | 0.06-32 | 0.125-256 | 0.125-256 |

MIC50, the minimal concentration inhibits 50% of isolates tested; MIC90, the minimal concentration inhibits 90% of isolates tested.

PEN, Penicillin; CRO, Ceftriaxone; TET, Tetracycline; ERY, Erythromycin.

Non-meningitis breakpoint: PEN resistant breakpoint, S ≤ 2 ug/ml, I = 4 ug/ml, R ≥ 8 ug/ml; CRO resistant breakpoint, S ≤ 1 ug/ml, I = 2 ug/ml, R ≥ 4 ug/ml.

Meningitis　breakpoint: PEN resistant breakpoint, S ≤ 0.06 ug/ml, R ≥ 0.12 ug/ml; CRO resistant breakpoint, S ≤ 0.5 ug/ml, I = 1 ug/ml, R ≥ 2 ug/ml.

TET resistant breakpoint, S ≤ 1 ug/ml, I = 2 ug/ml, R ≥ 4 ug/ml; ERY resistant breakpoint, S ≤ 0.25 ug/ml, I = 0.5 ug/ml, R ≥ 1 ug/ml.

‘*’, no isolate was obtained from sample hz21002 for AST, WGS was conducted directly to the blood sample.

**Supplementary Table 4.** The MIC value (mg/L) of non-invasive *S. pneumoniae* serotype isolates against tested antibiotics.

| **ID** | **PEN** | **CRO** | **TET** | **ERY** |
| --- | --- | --- | --- | --- |
| sy14004 | 0.06 | 0.125 | 16 | >128 |
| sy19035 | 0.125 | 0.125 | 32 | 128 |
| CH0002 | 0.125 | 0.06 | 64 | >128 |
| CH0025 | 0.125 | 0.06 | 32 | >128 |
| CH0047 | 0.06 | 0.06 | 32 | >128 |
| CH0051 | 8 | 4 | 32 | >128 |
| qz21031 | <0.06 | <0.06 | 64 | >128 |
| qz22014 | 4 | 0.5 | 64 | >128 |
| qz22024 | 4 | 2 | 32 | >128 |
| CH0009 | 0.125 | 0.06 | 8 | >128 |
| CH0017 | 4 | 0.5 | 32 | >128 |
| CH0058 | 4 | 4 | 4 | >128 |
| dy20008 | 0.12 | 0.25 | 32 | >128 |
| jh21030 | <0.06 | 0.12 | 0.125 | >128 |
| qz20016 | 0.125 | 0.25 | 2 | >128 |
| qz20045 | <0.06 | <0.06 | 64 | >128 |
| qz22004 | 2 | 1 | 16 | >128 |
| qz22022 | 2 | 1 | 4 | >128 |
| hz11012 | 0.125 | 0.125 | 16 | 128 |
| sy14022 | 2 | 0.5 | 16 | >128 |
| sy14023 | 2 | 0.25 | 16 | >128 |
| hz21006 | >32 | >32 | >32 | >128 |
| qz20024 | 2 | 1 | 32 | >128 |
| qz20041 | 4 | 1 | 64 | >128 |
| qz21024 | <0.06 | <0.06 | 32 | >128 |
| qz22006 | 4 | 1 | 32 | >128 |
| sy15002 | 4 | 0.5 | 8 | >128 |
| sy19002 | 4 | 0.5 | 16 | >128 |
| CH0037 | 8 | 4 | 16 | >128 |
| jh20031 | 0.25 | <0.06 | 16 | 8 |
| qz20011 | 0.5 | 0.25 | 32 | >128 |
| qz21017 | 1 | 0.5 | 128 | >128 |
| qz21028 | 0.5 | 0.5 | 0.25 | 64 |
| jx09002 | 0.06 | 0.125 | 8 | >128 |
| qz09009 | 0.06 | 0.125 | 8 | 0.06 |
| jx10011 | 0.06 | 0.125 | 0.06 | 0.06 |
| jx10012 | 0.125 | 0.125 | 16 | >128 |
| sy15003 | 0.125 | 0.125 | 16 | >128 |
| sy15005 | 0.125 | 0.125 | 16 | >128 |
| sy16013 | 0.06 | 0.125 | 16 | >128 |
| CH0027 | 0.125 | 0.015 | 32 | >128 |
| CH0038 | 8 | 4 | 32 | >128 |
| dy19008 | <0.06 | <0.06 | 16 | >128 |
| qz20043 | <0.06 | <0.06 | 64 | >128 |
| qz21003 | <0.06 | <0.06 | 64 | >128 |
| qz22027 | <0.06 | <0.06 | 64 | >128 |
| qz20012 | <0.06 | <0.06 | 0.25 | >128 |
| sy14018 | 0.06 | 0.125 | 4 | >128 |
| sy15031 | 0.06 | 0.125 | 4 | >128 |
| sy17007 | 0.06 | 0.125 | 0.25 | >128 |
| jh21048 | <0.06 | <0.06 | <0.06 | >128 |
| qz21004 | <0.06 | <0.06 | 1 | >128 |
| qz21012 | <0.06 | <0.06 | 16 | >128 |
| qz21018 | <0.06 | <0.06 | 8 | >128 |
| sy16029 | 0.06 | 0.125 | 0.125 | 8 |
| tz09007 | 0.125 | 0.125 | 8 | >128 |
| sy14017 | 0.06 | 0.125 | 0.125 | >128 |
| hz10016 | 0.06 | 0.125 | 32 | >128 |
| dy19018 | <0.06 | <0.06 | 32 | >128 |
| tz09011 | 0.06 | 0.125 | 4 | 0.06 |
| jx10005 | 0.125 | 0.125 | 32 | >128 |
| qz10017 | 0.06 | 0.125 | 0.06 | >128 |
| hz11001 | 0.125 | 0.125 | 4 | >128 |
| hz11005 | 1 | 0.125 | 32 | >128 |
| sy14019 | 0.06 | 0.125 | 0.125 | >128 |
| sy17011 | 0.06 | 0.125 | 0.125 | >128 |
| hz17003 | 0.125 | 0.125 | 32 | >128 |
| hz17005 | 0.125 | 0.125 | 32 | >128 |
| dy18006 | <0.06 | <0.06 | 128 | >128 |
| sy19037 | 0.125 | 0.125 | 32 | >128 |
| qz21010 | 0.125 | 0.125 | 4 | >128 |
| **MIC 50** | 0.125 | 0.125 | 16 | >128 |
| **MIC 90** | 4 | 1 | 64 | >128 |
| **MIC range** | 0.06-32 | 0.06-32 | 0.125-256 | 0.125-256 |

MIC50, the minimal concentration inhibits 50% of isolates tested; MIC90, the minimal concentration inhibits 90% of isolates tested.

PEN, Penicillin; CRO, Ceftriaxone; TET, Tetracycline; ERY, Erythromycin.

Non-meningitis breakpoint: PEN resistant breakpoint, S ≤ 2 ug/ml, I = 4 ug/ml, R ≥ 8 ug/ml; CRO resistant breakpoint, S ≤ 1 ug/ml, I = 2 ug/ml, R ≥ 4 ug/ml.

Meningitis　breakpoint: PEN resistant breakpoint, S ≤ 0.06 ug/ml, R ≥ 0.12 ug/ml; CRO resistant breakpoint, S ≤ 0.5 ug/ml, I = 1 ug/ml, R ≥ 2 ug/ml.

TET resistant breakpoint, S ≤ 1 ug/ml, I = 2 ug/ml, R ≥ 4 ug/ml; ERY resistant breakpoint, S ≤ 0.25 ug/ml, I = 0.5 ug/ml, R ≥ 1 ug/ml.
