# Supplementary figures and images for: Integrated genomic analysis of antibiotic resistance and virulence determinants in invasive strains of Streptococcus pneumoniae
Source: Front Cell Infect Microbiol. 2023 Oct 19;13:1238693. doi: 10.3389/fcimb.2023.1238693 (PMC10620807; doi:10.3389/fcimb.2023.1238693)

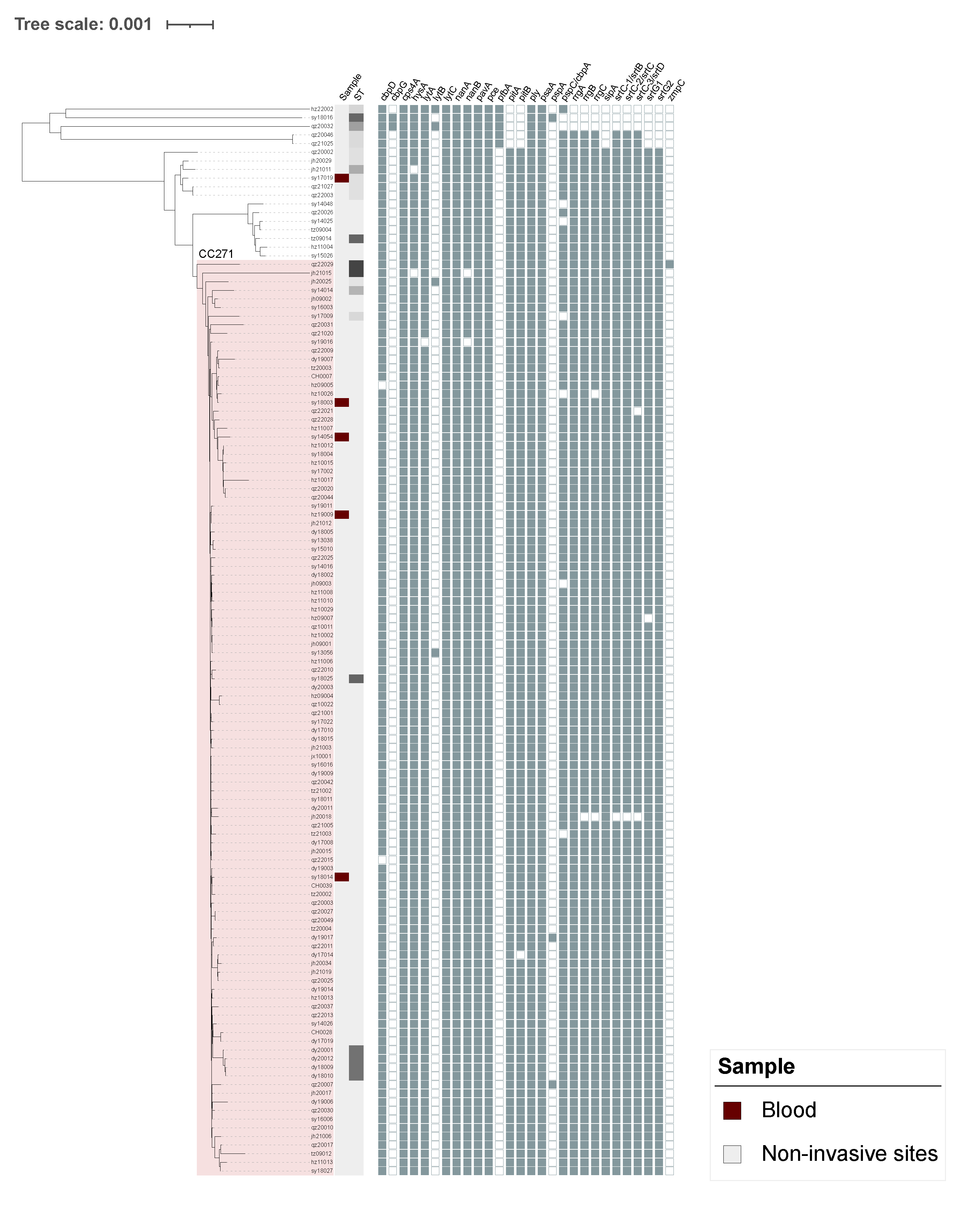

Supplement: Supplementary Figure 1 — The phylogenetic tree and antibiotic resistance determinants of pneumococcal serotype 19F strains. The phylogenetic tree of all sequenced pneumococcal serotype 19F isolates (n=124) was constructed in PopPUNK, where the major clone complex (CC) 271 was shaded in light red. The metadata including specimen types, sequence type (ST), antibiotic susceptibility test results (AST), and PBPs types were aligned for all isolates. [file Image_1.tiff]

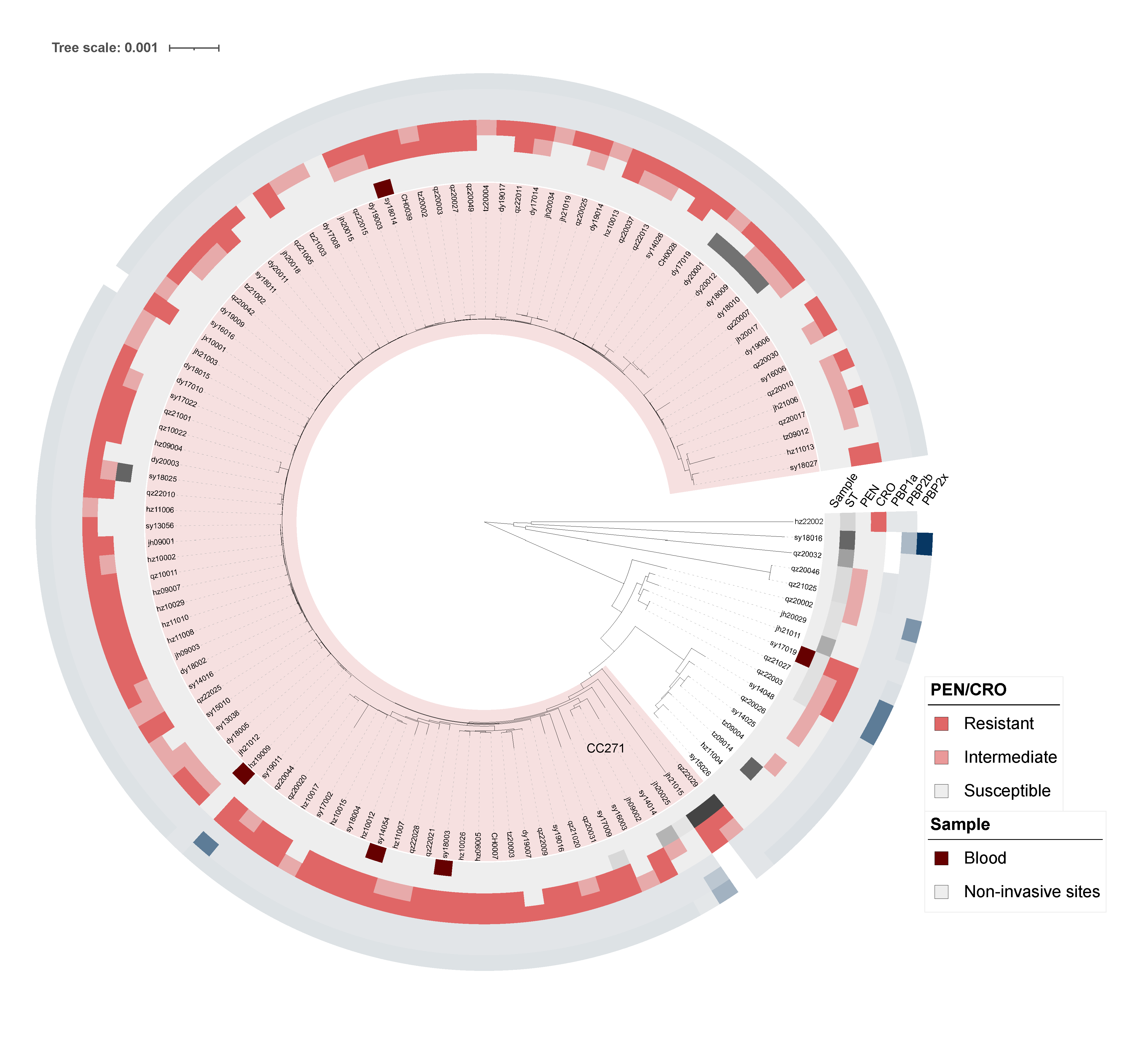

Supplement: Supplementary Figure 2 — Virulence factor detection in serotype 19F strains. The detection of virulence factors was attached for all isolates in the phylogenetic tree along with specimen data and sequence type of pneumococcal serotype 19F strains (n=124). A similar virulence factor carrying pattern was noticed in this clone. [file Image_2.tiff]
